# Current concepts in tumour-derived organoids

**DOI:** 10.1038/s41416-020-0993-5

**Published:** 2020-07-30

**Authors:** Ross J. Porter, Graeme I. Murray, Mairi H. McLean

**Affiliations:** 1 School of Medicine, Medical Sciences and Nutrition, University of Aberdeen, Scotland, UK; 2 Centre for Inflammation Research, Queen’s Medical Research Institute, University of Edinburgh, Scotland, UK

**Keywords:** Cancer models, Cancer models

## Abstract

Cancer comprises a collection of highly proliferative and heterogeneous cells growing within an adaptive and evolving tumour microenvironment. Cancer survival rates have significantly improved following decades of cancer research. However, many experimental and preclinical studies do not translate to the bedside, reflecting the challenges of modelling the complexities and multicellular basis of human disease. Organoids are novel, complex, three-dimensional ex vivo tissue cultures that are derived from embryonic stem cells, induced pluripotent stem cells or tissue-resident progenitor cells, and represent a near-physiological model for studying cancer. Organoids develop by self-organisation, and can accurately represent the diverse genetic, cellular and pathophysiological hallmarks of cancer. In addition, co-culture methods and the ability to genetically manipulate these organoids have widened their utility in cancer research. Organoids thus offer a new and exciting platform for studying cancer and directing personalised therapies. This review aims to highlight how organoids are shaping the future of cancer research.

## Background

Cancer mortality rates have significantly declined by ~26% over the past two decades,^1^ a decrease that is attributable to early diagnosis and treatment of malignancy, evidence-based clinical pathways for surveillance and management of premalignant lesions, increased awareness of health-related behaviours such as smoking and clinically focused cancer research. In spite of this success, however, cancer is the most common cause of death in the United Kingdom, and is expected to continue to remain as such, with 212,546 cancer deaths predicted for 2035.^2,3^

**Fig. 1:**
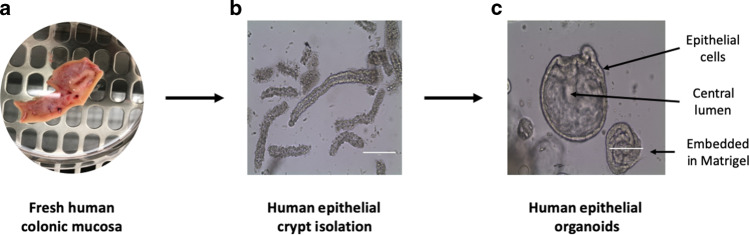
Establishing patient-derived organoid cultures. Patient-derived organoids reflect the genotype and phenotype of the original tissue, with preserved cellular heterogeneity and structural architecture. The critical steps involved in establishing a colonic organoid culture are **a** fresh colonic mucosa is obtained from human specimens (e.g., by biopsy or surgical resection), **b** colonic Lgr5^+^ stem cell-containing crypts are isolated and embedded in a basement membrane matrix, such as Matrigel and **c** colonic organoids are cultured in conditioned media containing specific growth factors and grow with a central lumen and representative apicobasal polarity. Images from Laboratory of Dr McLean, University of Aberdeen.

As we continue to make progress towards a ‘cure for cancer’, it is apparent that the data from many experimental and preclinical studies do not to translate from bench to bedside,^4,5^ an observation that is thought to reflect the challenges of modelling the complexities and multicellular basis of human disease.^6^ Despite these challenges, several pivotal systems, such as two-dimensional (2D) cell cultures, explants, organ-on-a-chip system and animal models, will continue to be essential to understand the biology of cancer. Nevertheless, novel and innovative model systems can improve the translational success of preclinical studies, and the methodology for tumour-derived organoid cultures has consequently emerged (Fig. 1). Organoids are complex, 3D, ex vivo tissue cultures that are derived from embryonic stem cells, induced pluripotent stem cells or tissue-resident progenitor cells. They possess spatially restricted lineage commitment and higher-order self-assembly, which makes them attractive near-physiological models.^7^ This review will firstly discuss the advantages and disadvantages of the experimental model systems that are currently used in cancer research, leading to a review of tumour-derived organoid model systems, including applications in cancer research, highlighting advantages, including potential utility in personalised medicine, limitations and future perspectives.

## Current experimental model systems in cancer research

**Fig. 2:**
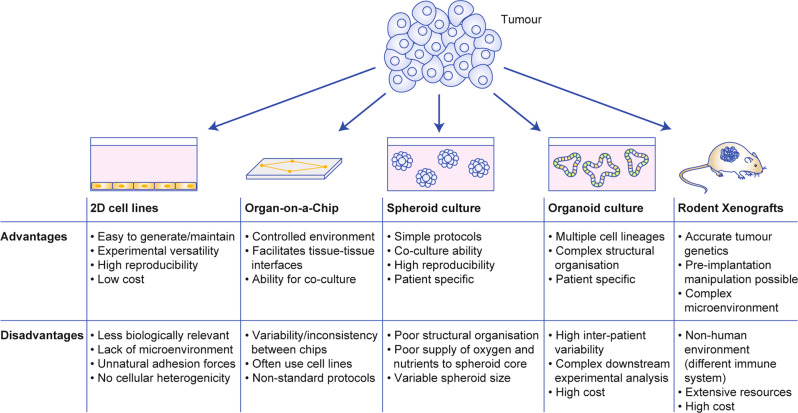
Advantages and disadvantages of model systems currently used in cancer research. There are several model systems used to study cancer in the laboratory. Using human tumours, the most common models are two-dimensional (2D) cell lines, organ-on-a-chip technology, spheroid cultures, organoid cultures and rodent xenografts, where human tumour is implanted into live animals. The advantages and disadvantages of these models are outlined here.

Several experimental model systems are currently applied to cancer research. Although a comprehensive overview of current laboratory models for cancer research is beyond the scope of this review, and is available elsewhere,^8–10^ the advantages and disadvantages are outlined in Fig. 2 and discussed below.

### 2D cell culture, tissue slices and tissue explant culture

2D cell cultures—either primary cell cultures (grown directly from patient or animal tumours) or well-characterised immortalised cell lines—have been extensively used to study cancer. Although primary cell lines have a limited lifespan and are slow-growing, they are advantageous because they maintain some donor-cell characteristics and can be linked to clinicopathological data. By contrast, artificial manipulation or natural genetic mutations confer on immortalised cell lines the ability to proliferate indefinitely, making them a more convenient and well-established preclinical model, but rendering them less representative of the original tumour; furthermore, serial passages induce genotypic and phenotypic changes that might confound experimental results.^11^ Irrespective of their origin, 2D cell cultures cannot replicate intra-tumour cellular heterogenicity, lack the complex extracellular microenvironment, have forced apicobasal polarity and are grown as a monolayer with unnatural suspension and adherence forces. Although co-cultures and transwell assays can address some of these issues, the biological translation of 2D cell culture models can be limited.

Tissue slices and organ cultures derived from tissue explants provide the architecture, morphology and cellular composition that 2D cell cultures lack. However, these models have a short lifespan (most tissues are viable for 24 h; the liver can be viable up to 96 h) and are expensive and difficult to maintain.^12^

### Organ-on-a-chip technology

Organ-on-a-chip technology refers to a multichannel microfluidic perfusion culture system, made from glass, plastic or a flexible polymer, that is lined with living human cells.^13^ This system allows more accurate modelling of organ-system physiology: for example, it facilitates the establishment of tissue–tissue interfaces, has separate vascular, extracellular and parenchymal compartments and allows for physiologically representative co-culture with microbes and immune cells.^14^ In cancer research, this technology has been used to study interactions between tumour cells and the extracellular milieu, cancer-associated epithelial–mesenchymal transition, angiogenesis, tumour invasion, cell migration and metastasis. Despite this impressive resume, this model does have disadvantages: for example, organ-on-a-chip platform commonly uses cell lines, and there is often significant variation and inconsistency between different chips, making experimental replication difficult.

### Animal models

Preclinical animal models continue to be a key aspect of cancer research, and these have been reviewed elsewhere.^15–20^ Tumour growth in vivo can be induced by chemicals (e.g., azoxymethane mouse model of colorectal cancer), viruses (e.g., Friend-virus-induced erythroleukaemia in mice) or radiation (e.g., UV radiation-induced melanoma in mice). Genetically engineered animals are popular because tumours can be induced to develop in transgenic mice (e.g., mice lacking the *adenomatous polyposis coli* [*APC*] gene are used to study the adenoma–carcinoma sequence in colorectal cancer) or knockout mice (e.g., the *BRCA1* conditional knockout mouse model using Cre/loxP recombination is used to study breast cancer), and key genes can be conditionally manipulated.^15,21^

Animal models are fundamental for translational cancer research—both for biological studies of pathogenesis and functional drug studies —and continue to be one of the cornerstone experimental approaches in the cancer research field. However, they do have limitations.^16,21^ Animal models are expensive, require extensive resources, and the data from many promising preclinical animal studies are often not validated in human models, or do not proceed in drug development towards clinical application,^21,22^ reflecting the different genetic, cellular and immunological characteristics in animals compared with humans. Steps taken to overcome these issues include transplanting human cancer tissue or cell lines into humanised rodents.^23,24^ These xenografts can be orthotopic (transplanted into the anatomical location from where the tumour was derived) or heterotopic (transplanted elsewhere, e.g., subcutaneously or intra-peritoneally).

## 3D cell models: tumour-derived organoid cultures

A critical player in organoid cultures is the stem cell, a self-renewing cell that can give rise to many different cell types within a tissue. Stem cells display unique markers such as leucine-rich repeat-containing G-protein-coupled receptor 5 (Lgr5) in the intestine. In 2009, Sato et al.^7^ reported that single-cell-sorted Lgr5^+^ stem cells located at the bottom of intestinal crypts can initiate crypt–villus organoids when embedded in Matrigel (a gelatinous protein matrix that provides the structural architecture to support 3D growth), and that these intestinal organoids contained differentiated cell types that are present in the original tissue.

It is important to distinguish organoids from spheroids: both are cultured in a 3D format, but spheroids are simpler, homogeneous, 3D structures that lack the multiple cell types seen in organoids. Spheroids typically represent free-floating cell aggregates with no matrix component—they usually depend on cell–cell adhesion for viability. Spheroids can be generated from immortalised cell lines, primary cells or fragments of tissue and, as such, their viability is limited as they do not contain a progenitor phenotype. Spheroids develop a necrotic core as they grow in size, and possess no or limited tissue structure and a less representative tissue architecture (e.g., no central lumen).^25^ Hence, although spheroid culture is a useful 3D culture methodology, offering a bridge between traditional 2D culture and costly in vivo animal studies, organoid-based 3D culture methodology offers several advantages to spheroids owing to enhanced architectural and physiological functions.

### Establishing and maintaining organoid cultures

The epithelial compartments of many tissues, including normal, premalignant tissues and tumours, have been modelled using organoids.^26^ Although the tissue for organoid culture is most commonly derived from surgical resection specimens,^27,28^ organoid cultures have been successfully established from other tissue sources, for example, endoscopic biopsy from Barrett’s oesophagus,^27^ needle biopsy for hepatocellular carcinoma,^29^ endoscopic ultrasound-scan-guided fine-needle biopsy for pancreatic ductal adenocarcinoma^30^ and ascitic fluid for both pancreatic and ovarian cancers.^25,31^ Successful tumour organoid cultures can therefore be generated from a small amount of biological material and from cancers that are difficult to access in the clinical setting. Previously, the interval between specimen collection and successful establishment of organoid culture has been dictated by the viability of fresh samples, which has limited the time, location and demographics from which a patient sample can be taken, but Tsai et al.^32^ published a robust method in 2018 to cryopreserve fresh human biopsy tissue and later thaw the specimen to generate gastrointestinal organoid cultures, thus overcoming this limitation. The diverse methods for tissue acquisition and the ability to cryopreserve specimens highlight the functional utility of organoid culture across a variety of cancer types and clinical situations.

Organoid cultures have been well-characterised in the literature, and this has provided a robust evidence base to validate the use of these models. For example, oesophageal adenocarcinoma organoids derived from oesophagectomy tissue specimens recapitulate the diverse genomic and transcriptomic landscape of the primary tumour,^33,34^ and histological assessment of these organoids demonstrated that the original tumour architecture and protein expression profile was maintained.^33^ This faithful representation has been reported across a variety of other tumour types, including, but not limited to, lung, ovarian, uterine, colorectal, bladder, liver, breast and biliary tract cancers.^29,34–38^ There is also evidence that epigenetic signatures in organoids appear to be reflective of those found in the primary lesion, indicating that the biology of the tumour is broadly represented.^39^

Once organoid cultures have been established, they require a complex and individualised combination of growth factors for survival and maintenance. It is essential to use optimised media formulations to ensure that experiments are reliable and reproducible. Organoid cultures from different tissues will have unique media requirements. Subtle changes to these cocktails can have marked consequences—for example, normal colonic organoids will outcompete colonic cancer organoids when cultured in media optimised for normal colonic organoids, potentially owing to apoptosis resulting from genomic instability in the tumour organoids.^35^ However, the sensitivity of organoids to growth factors can be exploited to establish many tumour organoid cultures. For example, normal colonic organoids require the ligand Wnt3a for survival, whereas the majority of colonic cancer cells demonstrate hyperactivation of the Wnt/β-catenin pathway independent of Wnt3a.^40^ Therefore, the selective removal of Wnt from organoid media prevents normal colonic organoids from outcompeting colonic cancer organoids.^35^ Not all colonic tumour cells display aberrant Wnt signalling though, and therefore, it is important to explore the implications of selecting tumour cells by their requirement(s) for specific factors.^41,42^ In future, it might be informative to use growth factor requirements to characterise, rather than select, tumour organoids. Nonetheless, it is important to remember that organoid function can be influenced by altering its media conditions, and it is therefore important to characterise organoid cultures before experimentation.

Long-term organoid culture is possible, with most groups reporting successful culture up to 6 months,^27,33^ and some groups reporting success beyond 1 year.^43^ Patient- and disease-specific characteristics are retained well over several passages.^43^ There is evidence that mutations do accumulate over time^33^ although this is perhaps unsurprising, given the known evolution of cancer in vivo,^44,45^ and this is consistent with tumour evolution in vitro.^36^ Tumour organoids also possess distinct organoid signatures that reflect real-life inter-patient variability.^46^ However, such inter-patient variability increases the sample-size requirements for robust power calculation-based experiments, which can be expensive. Ultimately, though, the expense must be weighed against the ability of this model to more accurately represent human disease.

## Advantages and limitations of organoid models in cancer research

**Fig. 3:**
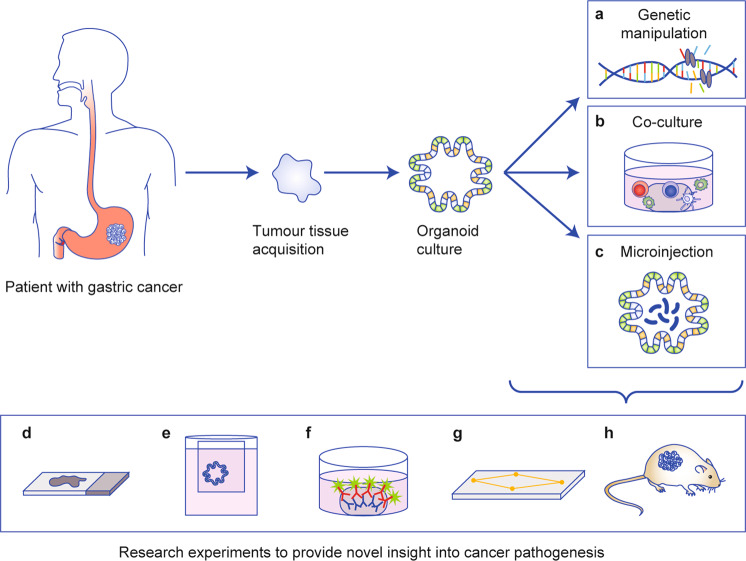
The use of patient-derived tumour organoids in cancer research. Tumour organoids can be manipulated to improve their functional utility for cancer research. They can be **a** genetically edited, for example, through CRISPR–Cas9 technology, **b** co-cultured with other cell types, such as immune cells, endothelial cells and stromal cells and **c** microinjected with microbes, antigens or chemicals. Several experimental approaches can be used to reveal novel insights into cancer pathophysiology, such as (**d**) immunohistochemistry (**e**), transwell and other 2D culture-based experiments (**f**) immunofluorescence, (**g**) organoid-on-a-chip technology and (**h**) xenografts.

Our ability to manipulate tumour organoids further improves the utility of this culture system in cancer research. Several experimental approaches can be used to reveal novel insights into cancer pathogenesis (Fig. 3).

### Tumour organoids enhance the utility of 2D cell culture

**Fig. 4:**
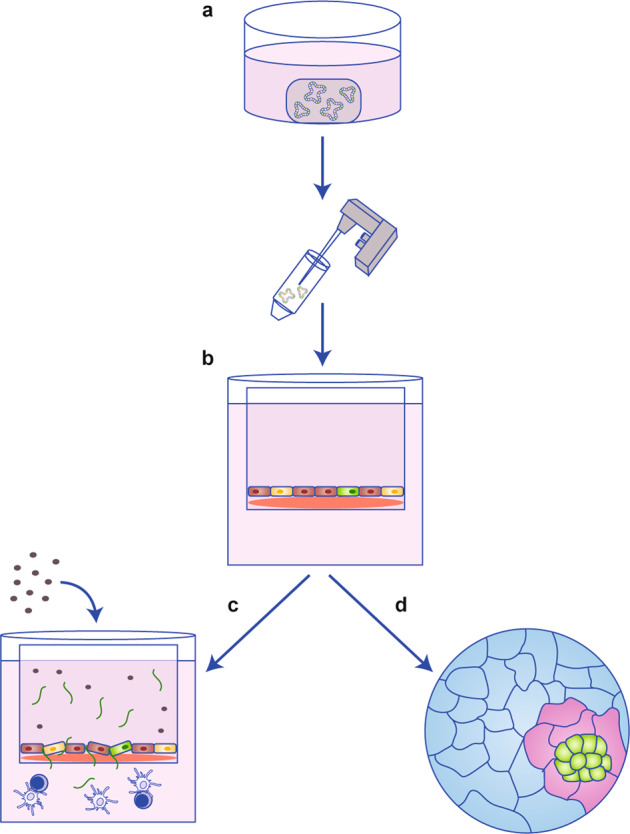
Organoid-derived 2D monolayer cultures can improve the versatility of organoids for cancer research. **a** Organoids can be grown in monolayer culture. **b** Transwell cell culture systems further improve the versatility of this model by allowing access to apical (luminal) and basal surfaces. **c** Transwell experiments are effective at exploring how luminal antigens or cytokines impact epithelial barrier integrity in cancer (i.e., through movement of fluorescein isothiocyanate–dextran or changes in transepithelial electrical resistance). Co-culture transwell experiments may further improve this model for cancer research (i.e., by investigating the response of T cells and dendritic cells to increased barrier permeability induced by luminal antigens/cytokines in cancer). **d** Using organoid-derived 2D monolayers allows more complex bioimage analysis to be performed.

As discussed above, 2D cell cultures constitute a key experimental platform in laboratory research—a myriad of validated experiments can be performed using these simple and inexpensive cultures. Consequently, the ability to establish 2D monolayers from epithelial-derived organoids allows functional experiments to be carried out, such as wound healing and transepithelial electrical resistance assays to measure functional permeability, while maintaining the unique characteristics of ex vivo organoid cultures, such as molecular identity to the original tissue and presence of a number of different epithelial cell types, such as parietal, chief and mucous neck surface mucosal cells from the stomach (Fig. 4).^47^ High-throughput microscopy can be performed in 2D-organoid-derived monolayer cultures, which would be difficult to perform in the 3D equivalent.

However, given the increased complexity of organoid models, there are some considerations for applying traditional 2D-based assays to 3D cultures. For example, organoid-derived monolayers cannot be easily passaged or propagated in 2D; instead, they achieve a homoeostatic state with balanced proliferation, differentiation and apoptosis.^48^ In addition, the efficiency of gene silencing using small-interfering (si)RNA is significantly reduced in the presence of serum in 2D cultures, whereas serum improves knockdown efficacy in organoid cultures by promoting the internalisation of siRNA.^49^

### Genome editing improves the functional utility of organoids for cancer research

Genome editing has been used to improve the use of organoid models and, as such, normal/non-cancerous organoid cultures can be genetically manipulated to undergo malignant transformation. The prokaryotic clustered regulatory interspaced short palindromic repeats (CRISPR)/CRISPR-associated protein 9 (Cas9) system has revolutionised how we approach genome editing in the laboratory, allowing precise and consistent in vitro genome editing.^50^ As an example, in human cerebral organoids, CRISPR/Cas9 technology has been used to facilitate the expression of an oncogenic HRas^G12V^ construct by homologous recombination into the *TP53* locus, thereby simultaneously disrupting this tumour-suppressor locus.^51^ This approach enables putative-initiating genetic mutations to be recreated, and the natural history of tumour initiation of human gliomas to be followed. CRISPR/Cas9 has also been used to study the origin of mutational signatures in cancer by the selective deletion of critical DNA-repair genes.^52^

By inserting an inducible histone 2B-green fluorescent protein (GFP) reporter into patient-derived organoid cells, CRISPR/Cas9 technology has been used to track quiescent versus proliferative cells during glioblastoma recurrence.^53^ Due to the ability of organoids to maintain tumour cell heterogenicity, this genetic manipulation allowed researchers to compare quiescent cells from glioblastoma tumours with their proliferative counterparts, which has revealed novel insights into the pathophysiology of glioblastoma. Indeed, quiescent cells were reported to differentially express genes involved in cell-cycle control, metabolic adaptation, interaction with the extracellular matrix and mesenchymal transition, and showed higher resistance to therapy compared with proliferative cells. Both hypoxia and transforming growth factor-β were identified as potential niche factors that promoted quiescence. This use of organoid technology has therefore laid the foundations for developing new therapeutic strategies for glioblastoma by uncovering novel mechanisms of recurrence for this tumour.^53^

To model the well-defined progression from adenoma to carcinoma that occurs during colorectal carcinogenesis, CRISPR/Cas9 has been used to genetically engineer sequential mutations associated with stem cell niche regulation, senescence and DNA mismatch repair in colonic organoids from mice to mirror the molecular pathogenesis of serrated colorectal cancer.^54^ Following genetic manipulation, the resulting requirement for growth factors in the stem cell niche was exploited to select for mutant organoids, and subsequent colonoscopy-guided orthotopic injection of organoids into mice was performed to investigate the contribution of specific mutations to carcinogenesis. The resultant tumours arising in vivo were reflective of human disease. This study demonstrates how genetically modified organoids can be used to create novel orthotopic models of cancer.

Genome editing in organoids has also revealed novel insights into the pathophysiology of previously hard-to-model diseases. Barrett’s oesophagus describes distal oesophageal columnar metaplasia with malignant potential. The disease is difficult to study because available cell lines and animal models are poorly representative of the underlying biology—cell lines lack the phenotypic diversity seen in the oesophageal submucosal glands, and mouse models do not accurately model neoplastic progression.^55,56^ Through the use of CRISPR/Cas9 technology, patient biopsy-derived Barrett’s organoids that lack *APC* have been generated and used to demonstrate a fundamental role of aberrant Wnt/β-catenin signalling in the neoplastic progression of Barrett’s-associated oesophagus.^57^

Alternative, less costly and time-consuming methods of genomic manipulation have also been reported in the context of gene editing. Such examples include lentiviral transduction of prostate epithelial organoids to demonstrate that genetic alterations that are commonly found in human prostate cancer can be modelled in human organoid culture,^58^ and magnetic nanoparticle viral transduction of gastrointestinal organoids for further study in in vitro assays or in vivo functional analyses in mice.^59^

### Organoids recreate the structural organisation of their origin tissue

Organoids can also recapitulate the spatial arrangement of their tissue of origin. When non-neoplastic bronchial mucosa failed to maintain its organoid ‘status’ during subculturing, Kim et al.^60^ added supplements known to promote lung development, which resulted in formation of bronchial cells; by passage 4, budding tubule-like organoids had emerged. Haematoxylin and eosin staining demonstrated that the organoids had pseudostratified epithelium, comprising basal cells and luminal cells that resembled normal bronchial mucosa. Interestingly, the non-neoplastic bronchial organoids had motile cilia found in large bundles, whereas lung cancer organoids had one single primary cilium per cell, indicating that, under the correct conditions, organoids can represent the structural organisation of their tissue of origin. In creating an organoid biobank from non-neoplastic and different subtypes of lung cancer tissue, the architectural, protein expression profile and molecular profiling was maintained from the tissue of origin.

Recreating structural morphology is especially important for cancers that arise from tissues with high structural organisation, such as the colon and rectum. Colorectal organoids self-organise to maintain apicobasal polarity with a hollow central lumen.^61^ This is a unique feature of organoid culture that reflects human anatomy and physiology, and it allows straightforward access to the basolateral surface via the culture media. Access to the luminal surface, however, is more difficult and, given the importance of antigens, microbiota and cell signalling receptors at the luminal surface of the epithelial barrier in colorectal carcinogenesis, there has been a focussed effort to improve luminal accessibility in organoid models. Breakdown of the epithelial barrier, with the consequent translocation of bacteria and luminal antigens through the colonic mucosa, is believed to be an important initiating event in colorectal carcinogenesis,^62^ and a protocol for the microinjection of fluorescently labelled dextran into patient-derived human intestinal organoids establishes a platform to study epithelial barrier dynamics in vitro.^61^ Breakdown of the epithelial barrier will result in increased permeability and translocation of fluorescently labelled dextran from the organoid lumen to the extracellular space. In addition to barrier integrity, the contribution of host–microbiota interactions to colorectal carcinogenesis can be studied by microinjection of live bacteria into the centre of colorectal organoids.^63^ Microinjection can be arduous and time consuming in the case of a large number of organoids. High-throughput microinjection of organoids is a solution to this problem, and can be achieved in the laboratory with semi-automated microinjection, microfabricated cell culture devices and computer vision systems (CVis).^64^ This technique could be applied to organoid cultures derived from various tumours, and could facilitate the injection not only of bacteria but also of chemical compounds, biological molecules, siRNA and other microorganisms.

One of the hallmarks of cancer is a loss of tissue organisation. Developments in microfabrication technology now enable organoids to be integrated into an extracellular matrix that contains specific biomolecules. For example, one study using normal breast organoid cultures created a defined chemoattractant gradient, using growth factors such as epidermal growth factor, to direct the formation of epithelial branches.^65^ This approach allows tissue-specific spatial and chemical factors to be considered in organoid cultures and, furthermore, can be adapted for use in cancer research—for example, to provide insights into factors that contribute to the loss of breast tissue organisation during carcinogenesis.

### The tumour microenvironment can be represented in organoid cultures using co-culture

Many laboratory findings fail to translate to the clinic because cell cultures do not accurately recapitulate cell behaviour and function within the wider tumour microenvironment, which includes the extracellular matrix, blood vessels, signalling components and other cell types. Consequently, organoids offer a biologically relevant platform to improve translatability. Co-cultures are not a new concept in the laboratory, as they are often used to study interactions between epithelial cells and other important cell populations, such as lymphocytes, neurones and blood vessels. The successful co-culture of epithelial cancer organoids with immune cells has revealed important insights into the pathogenesis of many cancers, and the ability to genetically manipulate such organoids with or without immune cells provides a specific and relevant model for studying carcinogenesis.^66–68^ Co-culture of mouse tumour organoids with adipocytes has provided novel insights into colon cancer. For example, Wen et al.^69^ demonstrate that adipocytes promote the proliferation and dedifferentiation (detected by increased Lgr5 and CD44, and decreased mucin 2 and sucrase–isomaltase mRNA levels) of colon cancer organoids. The authors further suggest that adipocytes function as a metabolic regulator and energy provider to promote the growth of colon cancer cells, which offers a potential mechanism to help explain the relationship between obesity and colorectal cancer.

The extracellular matrix is not a passive bystander in cancer biology; however, the biological consequences of this are often not explored or adjusted for in traditional laboratory experiments.^70^ Co-culture experiments can overcome this. For example, established pancreatic ductal adenocarcinoma organoids normally develop ductal and basement membrane structures, but this organisation is lost following co-culture with pancreatic stellate cells in a collagen matrix, coincident with basement membrane destruction and increased invasion into the collagen matrix.^71^ Furthermore, co-culturing pancreatic cancer organoids with both stromal and immune cells leads to the activation of myofibroblast-like cancer-associated fibroblasts, an observation that was not apparent in 2D culture models.^25^ A model system that allows interaction between cancer cells, stromal cells and immune cells is therefore important for studying the pathogenesis of cancer.

As well as making organoid cultures more representative of the in vivo scenario, co-culture can improve the differentiation yield. For example, co-culture of human-induced pluripotent stem cells with human adipose microvascular endothelial cells leads to an increased yield of hepatocyte-like clusters and the generation of hepatocyte-like organoids that resemble mature tissue rather than cell cultures.^72^ There is also evidence that adding primary prostate stromal cells to 3D cultures of human prostate epithelial cells increases the formation of prostate organoids and non-random architectural organisation in the form of branching.^73^

Techniques to improve organoid cultures continue to emerge, such as the use of self-generating hydrogels comprising extracellular matrix derived from human tissue instead of Matrigel. For example, Mollica et al.^74^ describe a method for generating extracts of mammary extracellular matrix that can spontaneously gel to form hydrogels. Importantly, these hydrogels retain biological signalling responses that are different between cancer and normal epithelial organoid cultures.^74^

Air–liquid interface systems, in which the basal surface of stem cells is in contact with the media and the apical surface is exposed to air, have also attracted interest. This set-up can more accurately reflect the conditions of the tumour microenvironment in certain cancers, such as the luminal surface of colorectal cancer.^75^ Usui et al.^75,76^ successfully developed air–liquid interface organoid models from normal and tumour colorectal tissues of human patients, and were able to demonstrate the presence of epithelial, goblet and fibroblast cells in normal colonic tissue, and epithelial, goblet, myofibroblast and cancer stem cells in colorectal cancer tissue, as well as to show that colorectal tumour organoids were more resistant to chemotherapeutic agents than colorectal cancer cell lines. Similarly, when investigating the effect of resistance to gemcitabine treatment, co-culture of pancreatic ductal adenocarcinoma organoids with cancer-associated fibroblasts resulted in an increased IC_50_ when compared with organoids cultured alone,^25^ indicating that organoid co-culture models can also offer novel insights into treatment responses. Ensuring a representative tissue microenvironment is therefore an important consideration for organoid culture in cancer research.

### Tumour organoids can enhance xenograft models

Patient-derived xenografts involve the implantation of human tissue or cells into humanised or immunodeficient rodents. This approach has provided invaluable insights into cancer invasion and metastasis; however, it can be improved further by the transplantation of organoids.^77^ Orthotopic transplantation is important to consider because subcutaneous xenografts often do not accurately recapitulate cancer invasion or metastasis.^78^ Orthotopic models of colorectal cancer have been developed from organoids and seen to produce uniform tumours that grow and metastasise reliably, depending on the metastatic potential of the cancer cells.^78^ One key example is the immunocompetent mouse model of colorectal cancer that recapitulates the well-defined human adenoma–carcinoma–metastasis sequence following orthotopic transplantation of colonic organoids.^79^ This approach can be applied to native or genetically modified human or mouse organoids: progression to adenocarcinoma occurs over 6 weeks, and spontaneous metastasis takes >20 weeks. Similar protocols use colonoscopy-guided mucosal injection and transplantation of organoids into the caecal mucosa of the mouse colon.^80,81^

A similar case exists in rectal cancer, for which there is a lack of anatomically relevant endoluminal rectal cancer mouse models. Ganesh et al.^82^ transplanted patient-derived rectal cancer organoids into mice, resulting in the generation of an invasive rectal carcinoma that metastasises to the liver and lung, as expected. Furthermore, the engrafted tumours display heterogeneous responses to chemotherapy, as also expected from clinical data.

Orthotopic transplantation can also take place after phenotypic and/or genotypic characterisation and/or manipulation of the tumour. For example, the CRISPR/Cas9 technology can be used to investigate the contribution of driver mutations in colorectal cancer.^83^ Such an approach could be used to generate the entire spectrum of cancer genotypes involved in carcinogenesis and metastasis,^84^ creating a biological library that can be used to investigate downstream phenotype changes, as has been reported.^85,86^ The insertion of a GFP tag by lentiviral transduction facilitates the straightforward detection of metastatic dissemination in such models.^87^

Cancers that develop from an orthotopically transplanted breast cancer organoid in mouse models not only reflect the morphology of the tumour of origin, but also the drug sensitivities, thereby rendering the ability to genetically modify such tumour-derived organoids invaluable in the study of drug resistance.^88^ Orthotopic transplantation overcomes many problems associated with other mouse models of colorectal cancer, such as a high tumour burden and tumours arising in the small intestine rather than the colorectum; as such, the use of organoids compared with cell culture clearly improves the translational ability of orthotopic transplant models.

## Future perspectives

### Tumour organoids can help replace, reduce and refine the use of animals in cancer research

Replacing, reducing and refining (3Rs) the use of animals in research is an international priority, and patient-derived organoid cultures represent an exciting platform to facilitate this principle. There are still limitations, such as the inability to mirror system-level interactions, multi-tissue interactions, multidirectional immune system interactions and explorations of drug pharmacokinetics and pharmacodynamics. However, these alternative organoid-based methods are evolving to study primary tumours ex vivo, and are adapting in complexity to overcome some of these limitations. As an example, metastasis adds a whole new dimension, and this process is difficult to study without the use of animal models. As discussed above, to overcome this, organ-on-a-chip technology constitutes an excellent animal-free model system for studying cancer metastasis, and can be improved by using organoid cultures. Aleman and Skardai^89^ have described a novel metastasis-on-a-chip system, in which colorectal cancer cells within a cancer organoid reside in a single microfluidic chamber that is connected to downstream chambers containing liver, lung and endothelial constructs in order to assess the metastatic preference of colorectal cancer cells. Other examples include the development of breast cancer-associated bone metastasis model^90^ and a multi-organ chip-based model of lung metastases with cell compartments representing bone, liver and brain.^91^ Organoids can therefore improve current disease models while helping to meet the international agenda outlined by the 3Rs.

Matrigel, which is currently widely used in the synthesis of organoids, is a basement membrane matrix with biological activity derived from Engelbreth–Holm–Swarm murine sarcomas.^92^ Animal-free alternatives, such as hydrogels made from alginates, do exist and have been used in novel model systems of the tissue microenvironment, such as a 3D bioprinted multicellular construct of breast tissue containing breast cancer cells and adipocytes,^93^ or utilising hyaluronic acid and collagen in a novel immersion bioprinting technique to allow organoid culture in 96-well plates for high-throughput drug screening, validated with patient-derived glioblastoma and sarcoma organoids.^94^

### Could tumour organoids inform patient management?

Organoids could be a future tool to facilitate decisions regarding patient management. As an example, patient-derived tumour organoids could help determine whether a particular patient will be sensitive or resistant to specific treatments for many cancer types in a personalised medicine approach.^95–98^ This knowledge could be especially useful when there are a lack of robust data from large randomised control trials, which is often the case for rare and metastatic cancers. For example, this approach has been explored using organoids from appendiceal,^99^ neuroendocrine prostate^100^ and sarcoma cancers^94^ to test the efficacy of various chemotherapeutic agents. Researchers in the Netherlands have also established colorectal cancer organoids from ascitic fluid and peritoneal metastasis and used them to assess sensitivity to chemotherapy agents in an in vitro hyperthermic intraperitoneal chemotherapy model.^101^ Consistent with variable clinical outcomes following hyperthermic intraperitoneal chemotherapy, the authors reported inter-patient variability in response to commonly used chemotherapeutics, suggesting that organoids could potentially allow treatment regimens to be individualised to improve prognosis and reduce rates of recurrence.

In addition to providing insights into individualised treatment responses, organoids could also help inform on drug toxicity.^102,103^ For example, rimonabant, a cannabinoid receptor 1 antagonist previously used in the management of obesity, which inactivates Wnt signalling and might therefore modulate cancer stemness in colorectal cancer, was shown to be selectively toxic towards colorectal cancer organoids but not healthy colonic cells, highlighting the potential use of rimonabant as a candidate for the treatment of colorectal cancer.^104^ The use of organoids for assessing response and toxicity to therapy is not restricted to chemical compounds; however, patient-derived rectal cancer organoids irradiated ex vivo were seen to display heterogeneous sensitivities that correlate with the patient’s clinical response to radiotherapy.^82^ Furthermore, Nagle et al. used organoids to demonstrate that proton irradiation carried out in a magnetic field did not impact biological responses.^105^

In addition to helping to select the correct therapy for patients, organoid models could also prevent cancer patients from receiving ineffective treatments. For example, in metastatic colorectal cancer, Ooft et al. demonstrated the use of patient-derived organoids in preventing patients from receiving ineffective irinotecan-based chemotherapy; interestingly, however, these patient-derived organoids were unable to predict the outcome for treatment with 5-fluorouracil plus oxaliplatin.^106^

This potential approach to personalised medicine has limitations. There can be a low success rate of generating some organoid cultures, probably dependent on tissue type. For example, Li et al. report an efficiency rate of 31% for generating oesophageal adenocarcinoma organoids.^33^ Success rates could be improved by employing tissue-quality evaluation protocols before culturing; this approach entails preparing and histologically examining an aliquot of cell suspension to select for epithelial cell-prominent samples following tissue dissociation.^60^

Patient-derived organoid culture could be useful in the study of chemotherapy resistance. When a patient becomes resistant to therapy, it might be possible to select for resistant cancer cells in culture by manipulating niche factor requirements, thereby facilitating screening for drugs that are effective against the resistant cells.^107^ High-throughput sequencing of organoid cultures is difficult, given that these cells are not likely to proliferate fast enough to generate the cells necessary for a very large screen. However, a bespoke, clinically relevant, drug screen could be performed on the resistant organoid culture to identify effective chemotherapy agents, and this approach appears more feasible at present. In addition, new methods to overcome this issue for high-throughput applications are emerging, including the use of bioprinting with alternative support matrix combinations to allow organoid culture in small-well culture plates, for example 96- or 384-well plates.^94^

Individualised cancer therapy based on ex vivo experiments using a patient’s own cancer organoids represents an ambitious goal for personalised medicine, and this approach is currently not achievable and is too expensive for most healthcare systems. However, organoid biobanks could represent a realistic intermediate step towards this goal. A gastric organoid biobank has been established and comprises 64 normal, dysplastic, cancer and lymph node metastasis organoids from 34 patients.^108^ This biobank includes most gastric cancer subtypes, and whole-exome and transcriptome analysis data are available for these cultures. Analysis has uncovered new understanding of cancer biology and disease pathogenesis. In addition, the utility of this organoid biobank is reflected in the results of the drug-sensitivity screen as this highlights the potential impact of drugs at a very early stage of development. A breast cancer organoid biobank of >100 primary and metastatic cancers,^38^ a lung cancer biobank^60^ and an ovarian cancer biobank of 56 organoids^109^ have generated similar results when used for in vitro drug screening. Across these studies, the organoid tumour biobanks remarkably maintain disease-specific subtype characteristics, such as morphology, transcriptomic profile and genomic mutational analysis to the native tumour even after long-term culture.^38,60,108,109^ Accordingly, they offer an exciting and realistic tool for precision medicine for use after the identification of a patient’s cancer subtype through histopathological identification with validated tumour biomarkers.

### Organoid cultures as novel treatment strategies

The immune response to tumours differs according to the biology of the underlying cancer. Autologous co-culture of tumour organoids derived from patients with colorectal cancer or non-small-cell lung cancer with peripheral lymphocytes leads to the expansion and enrichment of tumour-reactive T cells.^110^ These T cells are able to recognise and kill autologous tumour organoids but, remarkably, ignore healthy autologous organoids. This method enables us to investigate the mechanisms that underlie patient responses to immunotherapy. Furthermore, this approach could facilitate the generation of T-cell populations that could be used for autologous T-cell transfer therapy.

More direct potential therapeutic applications of organoids exist. Schwartz et al. have demonstrated that airbrush spraying of intestinal organoids onto a decellularised native extracellular matrix leads to the formation of an epithelial monolayer that resembles the intestinal surface.^111^ This is an exciting but underdeveloped concept that could have therapeutic benefits—for example, to help re-epithelialise areas affected by radiotherapy.

Radical surgery for breast cancer is often accompanied by radiotherapy or lymph node dissection. These adjuvant or neoadjuvant therapies are lifesaving, but increase the risk of life-long complications such as lymphoedema. In 2019, Lenti et al. investigated the transplantation of lympho-organoids into the region of dissected lymph nodes in mice.^112^ The lympho-organoids became fully integrated into the endogenous lymphatic system and restored lymphatic drainage. Furthermore, upon immunisation, the lympho-organoids were able to support antigen-specific endogenous immune responses. Therefore, therapeutic injection of lympho-organoids could become a novel therapeutic strategy for patients following radiotherapy or lymph node dissection for breast cancer.

## Conclusions

**Fig. 5:**
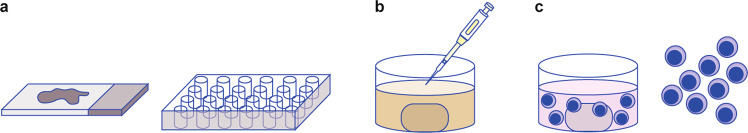
Bench to bedside: patient-derived tumour organoids could facilitate personalised medicine. Organoids can help direct personalised medicine through **a** using immunohistochemical markers to subtype cancers and predicting tumour response to specific therapies via biobank data, **b** by predicting an individual’s response to a specific therapy by organoid culture using tumour-derived organoids. **c** Future applications of organoids may involve a more direct role in treatment, such as using organoids to generate autologous tumour-reactive T cells.

Tumour-derived organoids are emerging as a tissue culture model that has exciting translational potential in the era of precision medicine. Tumour-derived organoids accurately represent the diverse genetic, molecular, morphological, architectural and functional pathophysiological hallmarks of cancer. Established cultures demonstrate intra-tumour and inter-patient heterogenicity, and can be further modified by genome editing, co-culture and orthotopic transplantation into rodents. In the clinic, tumour-derived organoids could be used to inform decisions on cancer treatment. In the wider setting, however, the translational impact of organoids is dependent on infrastructure, appropriate skill set and funding and, whilst the use of organoids is limited for now, there is future potential for this methodology and application to translate into clinical practice (Fig. 5). Overall, this cutting-edge method continues to evolve to provide new insights into the pathogenesis and evolution of cancer, offering the opportunity to develop new treatment strategies and enhance the impact of cancer research.

## Data Availability

Not applicable.
